# Perceptions and utilization of tele-orthodontics: a survey of the members of the American Association of Orthodontists

**DOI:** 10.1186/s40510-024-00516-4

**Published:** 2024-05-06

**Authors:** Sarah Abu Arqub, Dalya Al-Moghrabi, Chia-Ling Kuo, Lucas Da Cunha Godoy, Flavio Uribe

**Affiliations:** 1 Department of Orthodontics, University of Florida, Gainesville, FL USA; 2 Department of Preventive Dental Sciences, College of Dentistry, Princess Nourah bint Abdulrahman University, Riyadh, Saudi Arabia; 3 Connecticut Convergence Institute for Translation in Regenerative Engineering, UConn Health, Farmington, CT USA; 4 Division of Orthodontics, Department of Craniofacial Sciences, University of Connecticut Health, Farmington, CT USA

**Keywords:** Tele-orthodontics, Perception, Applications, Limitations

## Abstract

**Objective:**

This study aimed to investigate orthodontists’ utilization and perceptions of tele-orthodontics.

**Materials and methods:**

A 30-item online survey was distributed to members of the American Association of Orthodontists (AAO). The questionnaire encompassed topics concerning the orthodontists’ utilization, perceptions, clinical applications, limitations, and concerns regarding tele-orthodontics. Descriptive statistics were employed, and comparisons between responses from users and non-users were conducted  using Wilcoxon rank-sum tests and Fisher’s exact tests.

**Results:**

152 members completed the survey, (response rate: 2.4%). More than two third of respondents (69.74%) were users of tele-orthodontics. Users were more aligned with the belief that tele-orthodontics facilitates effective communication (mean ± standard deviation (SD) 4.06 ± 0.83 vs. 3.33 ± 0.94, p < 0.001). Both groups agreed on the requirement of patient fees for tele-orthodontic visits (mean ± SD: 3.62 ± 1.11 users vs. 3.74 ± 1.02 non-users, p = 0.659), and on the capability  of the system to reduce unwarranted referrals (p = 0.20). The majority of participants acknowledged  the utility of the system in monitoring aligners’ patients (89% in users vs. 61% in non-users, p < 0.001). Non-users expressed greater concerns regarding privacy risks (mean ± SD: 3.06 ± 0.97 users vs. 3.57 ± 0.86 non-users, p = 0.002). Both groups stressed the significance of obtaining informed consent before utilizing tele-orthodontics.

**Conclusions:**

The widespread acceptance of tele-orthodontics among AAO members was apparent, as demonstrated by their recognition of its effectiveness. There was notable variation in how users and non-users perceived tele-orthodontics. The study's results offer valuable insights into both the potential benefits and drawbacks of incorporating this technology into clinical practice from the clincians' perspective.

## Background

Tele-orthodontics is the application of information and communication technology along with the Internet to facilitate tele-consultation, monitoring, and continuing education in clinical orthodontics [1]. It is a form of tele-dentistry that implements tele-communications in orthodontic practices. The concept of tele-dentistry was initially developed in 1994 as part of a military initiative for the United States Army (U.S. Army’s Total Dental Access Project) aimed to enhance communication between dental specialists and laboratories, and enhance the standards of patient care and dental education [2]. Over the years, tele-dentistry in general and tele-orthodontics in specific have proven to be effective in facilitating patient-provider communication, especially during the COVID-19 pandemic [3–8]. Thereafter, its popularity has significantly increased. Further, tele-dentistry has been proven to aid in the interceptive orthodontic treatment of children in lower socioeconomic groups who have limited access to orthodontic services [9]. Tele-orthodontics can be cost-effective, potentially reducing unnecessary visits, and enabling remote monitoring, particularly for clear aligner patients [10]. Moreover, tele-dentistry offers real-time consultations and data storage, facilitating discussions between clinicians and improving access to care [11]. However, considerations related to ethical concerns and breaches in confidentiality due to the exchange of sensitive information online must not be overlooked [12].

The American Dental Association (ADA) categorizes tele-dentistry into key modalities: (1) synchronous, which includes real-time video-audio communication; (2) asynchronous, which includes secure exchange of saved information such as photographs, videos, X-rays among clinicians; (3) Remote Patient Monitoring (RPM), which collects and transmits personal health-related data, and (4) Mobile Health (mHealth), which utilizes mobile communication for education, public health, and practices [13]. In the field of clinical tele-orthodontics, communication between clinicians and patients can encompass video calls, consultations for clear aligner therapy, or sharing of clinical photographs and instant messaging [14]. These current treatment monitoring systems are well-received by practitioners who reported optimism about integrating tele-dentistry into their practices[15]. Patients also reported positive experiences and expressed willingness to continue using the system post-pandemic due to time-saving benefits [16]. Furthermore, Mandall et al. [17, 18] reported the importance of a tele-dentistry for screening new patients, managing orthodontic referrals, and reducing inappropriate referrals.

To our knowledge, all the previous studies thus far have assessed the efficacy of tele-dentistry and its applications in a general dental practice set up [1, 4, 6, 7, 9], with none specifically exploring its usefulness for the orthodontic practice. Therefore, the aim of this cross-sectional study was to explore the utilization and perceptions concerning the efficacy of tele-orthodontics among American Association of Orthodontists (AAO) members; and to highlight the clinical applications, limitations and concerns related to tele-orthodontics. Furthermore, users and non-users of tele-orthodontics were compared regarding demographics, clinical experience, and perceptions regarding tele-orthodontics.

## Materials and methods

The protocol for this cross-sectional survey study was approved by the ethical committee of UCONN Health IRB number: 22X-231-1. A comprehensive evaluation of the literature in relation to efficacy, usefulness, and future applications of tele-orthodontics in clinical practice was carried out to develop the survey instrument. An initial draft for the survey included 39 questions and was constructed using Google forms (online survey tool). These questions were evaluated for content validity by 12 consultant orthodontists who were clinical professors in academic institutions in the United States, with more than 10 years of clinical experience. The “Lawshe’s Method” was implemented to calculate Content Validity Ratio (CVR) for each question [19]. This method is based on evaluating each question based on a 3-point scale (not necessary, useful non-essential, and essential) [19]. Nine questions were found non-significant at a critical level according to “Lawshe’s Method”, thus, were excluded from the final version of the online survey.

The final survey instrument consisted of 30 questions and covered the following sections: demographics, use of tele-orthodontics, orthodontists’ utilization and perspectives of usefulness and efficacy of tele-orthodontics for patients and clinical practice, as well as the clinical applications and limitations of tele-orthodontics, concerns about security, confidentiality, and consent (Appendix). The surveylength.com instrument calculator was used to ensure a reasonable time for the survey completion based on the number and type of question, in addition to the age of the respondents. The estimated time calculated for survey completion was 10 min. The review committee of the AAO Partners in Research program, reviewed and edited the questionnaire. Upon approval, the final survey instrument was distributed via e-mail to the active members of the AAO in the United States. The survey was distributed on three occasions to a randomly selected sample of members (n = 2100, 2113 and 2111), with a reminder e-mail sent after each distribution. The response rate was calculated by dividing the number of respondents by the total number of contacted members (n = 6324).

### Statistical analysis

Respondents were grouped into users and non-users of tele-orthodontics based on their responses to survey question #6 (Appendix). Descriptive statistics using mean and standard deviation (SD) as well as frequencies and percentages were performed. Responses from free-text were coded, and frequencies were determined. Numerical scores for Likert-scale questions were compared between users and non-users of tele-orthodontics using Wilcoxon rank-sum tests.

Five-item Likert-scale responses were scored from 1 to 5, from strongly disagree to strongly agree. Reversely, efficiency of tele-orthodontics’ appointments was ranked by responders from 1 to 5, most efficient to least efficient. The numerical scores per question were summarized by mean ± SD, median and range, and they were compared using Wilcoxon rank-sum tests between users and non-users of tele-orthodontics, based on their responses to the survey question, “I started using tele-orthodontics in my practice”: “have not used all” as *non-users*; “during the pandemic (first 3–6 months until currently)”, “pre-pandemic”, or “during the pandemic (6 months until currently)” as *users*. The responses of users and non-users were also compared using Fisher’s exact tests for demographics and perceptions of tele-orthodontics that could not be processed to numerical scores. Questions that allowed the responders to “tick all that apply” were analyzed option-wise. P-values less than 0.05 were considered statistically significant. Missing data were excluded from analysis. All the hypothesis tests were two-sided, and the statistical analyses were performed using R version 4.3.1.

## Results

A total of 152 active members from the AAO responded to the distributed online survey between November 2022 and April 2023. The response rate was 2.4%. The responders were divided into users of tele-orthodontics (69.74%), and non-users of tele-orthodontics (30.26%) (Table 1). The mean age of the participants was 52 (standard deviation (SD): 14) years for non-users compared to 55 (SD: 11) years for users (p = 0.266). The majority of the responders were males (78.26% in non-users vs. 68.87% in users), and had greater than 20 years of clinical experience (54.35% in non-users vs. 65.1% in users). Private practitioners constituted the bulk of the responders in both groups. The majority of the users (57.55%) indicated that they started using the system in the first 3–6 months of the pandemic. Video calls was the most preferred tele-orthodontic communication protocol among users (60.38%). Interestingly, responders indicated the use of other up-to-date means of interaction systems, among which Dental Monitoring (DM; Paris, France) system was the most commonly used.

**Table 1:** Demographics and general information (up to 152 respondents)

Question (number of respondents)	Non-users of tele-orthodontics (N = 46)	Users of tele-orthodontics (N = 106)	*p-*value
Age (n = 151)	Mean ± SD: 52 ± 14 years	Mean ± SD: 55 ± 11 years	0.266
Gender (n = 152)			0.327
Female	10 (21.74%)	33 (31.13%)	
Male	36 (78.26%)	73 (68.87%)	
Years of clinical experience (n = 152)			0.026
Less than 5 years	7 (15.22%)	7 (6.54%)	
5–10 years	9 (19.57%)	7 (6.6%)	
10–15 years	1 (2.17%)	11 (10.38%)	
15–20 years	4 (8.7%)	12 (11.32%)	
More than 20 years	25 (54.35%)	69 (65.1%)	
Society of practice (n = 143)			0.846
GLA	4 (9.3%)	13 (13%)	
MAS	5 (11.63%)	13 (13%)	
MWS	4 (9.3%)	17 (17%)	
NES	4 (9.3%)	10 (10%)	
PCS	10 (23.26%)	14 (14%)	
RMS	3 (7%)	7 (7%)	
SAO	10 (23.3%)	21 (21%)	
SWS	3 (7%)	5 (5%)	
Type of practice (n = 152)			0.007
Academic	3 (6.52%)	5 (4.71%)	
Corporate	11 (23.91%)	7 (6.6%)	
Private practice (one orthodontist)	24 (52.17%)	55 (51.88%)	
Private practice (two or more orthodontists)	8 (17.39%)	39 (36.8%)	
Initiation of tele-orthodontics utilization (n = 152)			< 0.001
Pre-pandemic	0 (0%)	25 (23.58%)	
During the pandemic (first 3–6 months until currently)	0 (0%)	61 (57.55%)	
During the pandemic (6 months until currently)	0 (0%)	20 (18.87%)	
Not used	46 (100%)	0 (0%)	
Preferred tele-orthodontic protocol (tick all that apply) (n = 150)			
Smile consult—align technology	5 (11.36%)	6 (5.66%)	0.301
Sharing photos and instant messaging	14 (31.81%)	53 (50%)	0.048
Video calls	11 (25%)	64 (60.38%)	< 0.001
Other applications	14 (31.82%)	28 (26.42%)	< 0.001

*GLA* Great Lakes Association, *MAS* Middle Atlantic Society, *MWS* Midwestern Society, *NES* Northeastern Society, *PCS* Pacific Coast Society, *RMS* Rocky Mountain Society, *SAO* Southern Association of Orthodontists, *SD* standard deviation, *SWS* Southwestern Society of Orthodontists

**Fig. 1:**
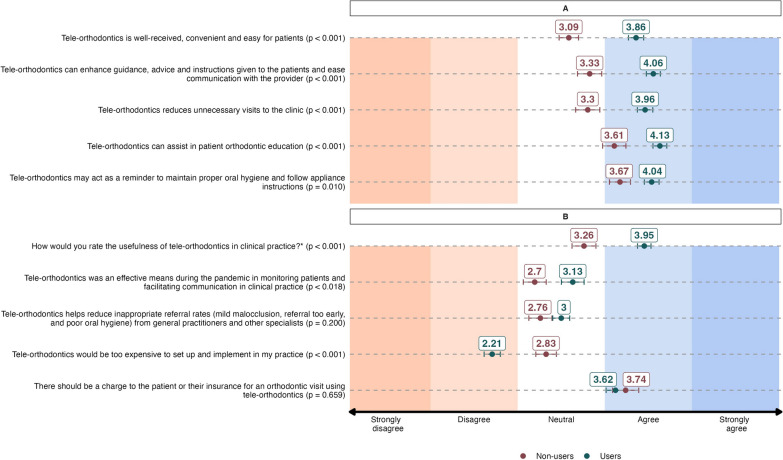
Orthodontists’ perception of usefulness and efficacy of tele-orthodontics for patients (**A**) and the clinical practice (**B**). *(1 extremely useful to 5 non-useful) and (1 strongly agree to 5 strongly disagree)

Users of tele-orthodontics had more positive views, reflected in higher mean scores regarding the potential benefits of the system compared to non-users (p < 0.05) (Fig. 1A). Users of tele-orthodontics assigned a higher mean score to the system’s convenience, reception and user-friendliness for the patients (3.86 ± 0.90) compared to non-users (3.09 ± 0.76) (p < 0.001). Additionally, users scored higher than non-users in agreement with statements related to tele-orthodontics facilitation of communication with the provider (4.06 ± 0.83 vs. 3.33 ± 0.94, p < 0.001), reduction of unnecessary visits (3.96 ± 0.91 vs. 3.30 ± 0.94, p < 0.001), assistance in patients’ orthodontic education (4.13 ± 0.82 vs. 3.61 ± 0.91, p < 0.001) and acting as a reminder for promoting oral hygiene practices (4.04 ± 0.87 vs. 3.67 ± 0.82, p = 0.010).

Users demonstrated higher agreement than non-users regarding the usefulness of tele-orthodontics in clinical practice (3.95 ± 0.80 in users vs. 3.26 ± 0.93 in non-users, p < 0.001) (Fig. 1B). Additionally, users assigned higher scores, indicating a significant benefit in providing an effective mean to communicate with patients during pandemic era (3.13 ± 1.34 in users vs. 2.70 ± 0.89 in non-users, p = 0.018). Conversely, non-users agreed more to the statement that implementing and setting up the system in their clinical practice would be too expensive (2.83 ± 0.80) compared to the users (2.21 ± 0.95) (p < 0.001). Both groups similarly agreed that there should be a charge to the patient for an orthodontic visit using tele-orthodontics (p = 0.659), and that the system might help reduce inappropriate referrals (p = 0.20).

More than half of the responders indicated that both adults and children can benefit from tele-orthodontics (69.87% in users vs. 52.17% in non-users) (Table 2). The majority of respondents indicated the usefulness of the system in primarily monitoring patients undergoing clear aligner therapy (88.68% in users vs. 60.87% in non-users, p < 0.001) and handling emergencies (76.42% in users vs. 60.87% in non-users, p = 0.077). The users of the system agreed more than non-users to the statements that the system that the system serves as an adequate diagnostic tool and provides screening capabilities for prospective patients (mean ± SD: 3.27 ± 1.15 in users vs. 2.43 ± 1.09 in non-users, p < 0.001), helps monitor the rate of tooth movement (3.84 ± 0.85 in users versus 3.09 ± 0.91 in non-users, p < 0.001) and can be an efficient tool in interceptive orthodontic treatment (3.29 ± 1.05 in users vs. 2.65 ± 0.97 in non-users, p < 0.001).

**Table 2:** Summary of responses concerning clinical applications of tele-orthodontics (up to n = 152 respondents)

Question	Non-users of tele-orthodontics (N = 46)	Users of tele-orthodontics (N = 106)	p-value
Patients that would benefit the most from tele-orthodontics			< 0.001
Adults	12 (26.09%)	30 (28.3%)	
Children	1 (2.17%)	1 (0.94%)	
Both	24 (52.17%)	73 (69.87%)	
Other	9 (19.57%)	2 (1.89%)	
Tele-orthodontics is mostly used in (tick all that apply)			
Fixed orthodontic appliances	10 (21.74%)	32 (30.19%)	0.328
Clear aligner therapy	28 (60.87%)	94 (88.68%)	< 0.001
Emergencies	28 (60.87%)	81 (76.42%)	0.077
Retainers	14 (30.43%)	54 (50.94%)	0.022
Other	2 (4.35%)	14 (13.21%)	0.002
Tele-orthodontics often provides sufficient diagnostic information and serves as a suitable screening tool^§^	2.43 ± 1.09 (2; 1–5)*	3.27 ± 1.15 (4; 1–5)*	< 0.001
Tele-orthodontics helps monitor rate of tooth movement and treatment progress^§^	3.09 ± 0.91 (3; 1–5)*	3.84 ± 0.85 (4; 1–5)*	< 0.001
Tele-orthodontics can be an effective tool for interceptive orthodontic treatment^§^	2.65 ± 0.97 (3; 1–5)*	3.29 ± 1.05 (3; 1–5)*	< 0.001
Rank the efficacy of telematic appointments from 1 (most effective) to 5 (least effective):			0.026
Screening, planning, and consultation	3.52 ± 1.31 (3; 1–5)*	2.74 ± 1.35 (3; 1–5)*	0.001
Delivery and monitoring of aligners	3.14 ± 1.27 (3; 1–5)*	2.67 ± 1.36 (2; 1–5)*	0.05
Delivery of elastics and their use	3.17 ± 1.23 (3; 1–5)*	2.92 ± 1.25 (3; 1–5)*	0.256
Follow-up appointments for visualization of tooth movement	2.96 ± 1.28 (3; 1–5)*	2.65 ± 1.23 (2; 1–5)*	0.176
Handling emergencies	2.66 ± 1.38 (3; 1–5)*	2.09 ± 1.13 (2; 1–5)*	0.017
There will be a significant increase in tele-orthodontics in the future^§^	3.41 ± 1.05 (4; 1–5)*	3.98 ± 0.89 (4; 1–5)*	0.002

*Data presented as mean ± standard deviation (median; range)

^§^Rated from 1 (strongly disagree) to 5 (strongly agree)

The group of users had significantly more favorable views than non-users regarding the perceived effectiveness of telematic appointments in screening, planning, and consultations (mean ± SD: 2.74 ± 1.35 in users vs. 3.52 ± 1.31 in non-users, p = 0.001), and in handling emergencies (2.09 ± 1.13 in users vs. 2.66 ± 1.38 in non-users, p = 0.017). Further, the group of users showed greater agreement with the statement predicting a significant increase in tele-orthodontics in the future (mean ± SD: 3.98 ± 0.89) compared to non-users (3.41 ± 1.05, p = 0.002).

Non-users had higher scores than users regarding concerns of implementing tele-orthodontics on patients’ compliance (mean ± SD: 3.34 ± 0.99 in users vs. 4.09 ± 0.89 in non-users, p < 0.001), and that patients with medical conditions and on medications should not be candidates for tele-orthodontics (mean ± SD: 3.25 ± 1.22 in users vs. 4.02 ± 1.06 in non-users, p < 0.001) (Table 3). Additionally, the non-users agreed more with the statement that the biomechanical concerns raise caution when using remote monitoring (mean ± SD: 3.49 ± 1.11 in users vs. 4.30 ± 0.70 in non-users, p < 0.001). Moreover, the non-users were more in agreement than users that sharing data online might violate patients’ privacy (mean ± SD: 3.06 ± 0.97 users vs. 3.57 ± 0.86 non-users, p = 0.002). Both groups agreed that obtaining informed consent is essential before the use tele-orthodontics. Furthermore, the majority of users (72.64%) indicated that tele-orthodontics is considered legal in their states while 63.04% of non-users were uncertain about its legal status.

**Table 3:** Summary of responses regarding limitations and concerns related to the use of tele-orthodontics (n = 152 respondents)

Question	Non-users of tele-orthodontics (N = 46)	Users of tele-orthodontics (N = 106)	p-value
Irregular in-person visits may influence patients’ compliance with oral hygiene and adherence to treatment protocols	4.09 ± 0.89 (4; 2–5)*	3.34 ± 0.99 (4; 1–5)*	< 0.001
Patients with systematic conditions, severe caries, periodontal disease, or medications jeopardizing orthodontic treatment should not be candidates for tele-orthodontics^§^	4.02 ± 1.06 (4; 2–5)*	3.25 ± 1.22 (3; 1–5)*	< 0.001
Biomechanical treatment concerns raise caution when using remote monitoring^§^	4.30 ± 0.70 (4; 3–5)*	3.49 ± 1.11 (4; 1–5)*	< 0.001
Tele-dentistry legal acceptability in the state of practice?			< 0.001
I don't know	29 (63.04%)	28 (26.42%)	
No	5 (10.87%)	1 (0.94%)	
Yes	12 (26.09%)	77 (72.64%)	
Tele-orthodontics may violate patients’ privacy and confidentiality when data is sent online^§^	3.57 ± 0.86 (4; 1–5)*	3.06 ± 0.97 (3; 1–5)*	0.002
Informed consent is necessary for tele-orthodontics, as sharing patients' photos and clinical information poses a risk to confidentiality^§^	4.22 ± 0.92 (4; 1–5)*	4.26 ± 0.83 (4; 1–5)*	0.767

*Data presented as mean ± standard deviation (median; range)

^§^Rated from 1 (strongly disagree) to 5 (strongly agree)

## Discussion

Based on the findings of this study, it is evident that the use of tele-orthodontics is prevalent among AAO members, with over two thirds reporting its utilization. Most users implemented the system during the pandemic, mainly for managing clear aligner patients and handling emergencies. This was mainly in the form of video calls, sharing photos and instant messages. The users of tele-orthodontics viewed the efficiency of the system in screening, planning and consultations and the potential increase in its use more positively than non-users. Non-users perceived the impact of tele-orthodontics on patient adherence more negatively. Both groups had concerns regarding patient safety and confidentiality with data sent online; however, this was higher in non-users. Both groups generally agreed about the need for informed consent prior to using the system.

System users indicated that video calls are the most preferred tele-orthodontic communication protocol (60.38%). A previous study found that patient satisfaction was highest with video consultations due to their ease, convenience, and associated positive experience [20]. In the current study, users of tele-orthodontics indicated that the system is convenient and user-friendly for patients, assists in patient education and promotes compliance with oral hygiene practices. Tele-orthodontics is technologically accessible and patient consultations can be conducted in a convenient environment without the need to commute [21]. Other advantages the system can provide for patients are related to time savings, reduced transportation costs and early detection of issues, such as bracket failures, broken retainers and non-tracking aligners [21]. These advantages might also positively affect the clinical environment, reducing the number of unnecessary appointments and the operating costs for an orthodontic practice and providing more efficient and timely treatment, increasing the patient starting pool and number of finished cases. Moreover, the perceived benefits for the system in aspects related to better communication with the provider and reduced number of required appointments [21]. A recent overview of nine moderate-to high-quality systematic reviews found that incorporating such systems in clinical practice plays a major role in enhancing patient education and compliance with oral hygiene practices, elastic use and the wear of removable appliances [22]. Overall, the system demonstrates promising benefits for orthodontic patient and practices.

Both groups of responders agreed regarding the additional charge that should be associated with providing this service to patients. This might be related to concerns regarding the costs associated with utilizing tele-orthodontic monitoring equipment. Therefore, preliminary investigations are needed to assess the cost-effectiveness of the clinical integrating tele-orthodontics [23]. Both groups agreed that inappropriate referral rates from general practitioners could be substantially reduced using of tele-orthodontics. This was in agreement with Mandall et al. who evaluated the efficacy and validity of tele-dentistry in screening orthodontic patients [11]. Further research and cost-effectiveness assessments are crucial to inform the incorporation of tele-orthodontics into clinical practice.

The broad scope of clinical applications of tele-orthodontics and its ease of use, made it a viable tool to monitor orthodontic patients undergoing different treatment modalities. Clinical applications might include screening, diagnosis and initial treatment planning. However, in-person evaluation remains the foundation for clinical diagnosis, and tele-orthodontics can be used for initial screening and consultations [24]. It must be noted that further evaluation is required concerning the reliability of the visual information obtained through tele-dentistry [25]. Furthermore, the inability to detect potential risk factors associated with orthodontic treatment, such as medical disorders and habits, is a potential drawback if the clinician relied only on tele-orthodontics for the diagnosis [26]. Hasna et al. demonstrated the added value of DM (Paris, France) to a group of patients treated with the Invisalign® (Align Technology, Santa Clara, Calif) system [27]. In their study, a significant reduction in the number of appointments by 3.5 visits (33.1%) and a reduction in the time to the first refinement (1.7 months) in the DM group [27]. Furthermore, tele-orthodontics can be useful in the retention follow-up visits and early interceptive treatment appointments, both of which require monitoring progress and compliance assessment rather than an activation procedure [24, 28].

The need for tele-orthodontics to resolve orthodontic emergencies was boosted during the COVID-19 pandemic. Arqub et al. found that 73% of orthodontic patients avoided in person visits and preferred phone calls or text messages to resolve emergencies [29]. Caprioglio et al. proposed guidelines for managing orthodontic emergencies via tele-orthodontics by classifying the emergencies into loose brackets, poking ligatures and distal wires, broken retainers and aligners [30]. In the current  cross-sectional study, both users and non-users of the system agree that tele-orthodontics is an efficient tool for troubleshooting and handling emergencies via various modes of telecommunication such as videos, images, and text messaging. Moreover, the system users were in greater agreement than non-users that the system can provide an efficient tool to monitor the rate of tooth movement. Clinical live tracking of the rate and amount of tooth movement is now feasible with the patient-managed smartphone application associated with the online doctors’ platform for DM (Paris, France). This technology can evaluate 3D tooth positions from superimposed original 3D models constructed from an intraoral scan on video-scans consequently taken by patients’ smartphones [31].

Since the utilization of remote monitoring might reduce the number of visits to the orthodontist’s office, the role of the clinician might become less relevant. Moreover, the patient-orthodontist relationship might be affected; therefore, compliance with appliance wear and oral hygiene practices might be impaired, as indicated by the concerns of the non-users in the current study. Studies have shown positive experiences and perceptions of patients using tele-orthodontics [21, 27]. Some tele-orthodontics applications allow direct messaging with the orthodontist, which compensates for the communication problems potentially resulting from the reduced number of visits [32]. However, patients might become confused and start seeking the “do it yourself” less expensive treatment option, erroneously believing that orthodontic treatment can be successfully implemented remotely [24]. Moreover, this survey has indicated that concerns might arise regarding the confidentiality of information due to the transfer of information via tele-orthodontics and storage of data on computers and online platforms [33]. Therefore, patient privacy should be secured, and patients should be informed that their information is transmitted electronically and may be intercepted [33]. This aligns with the findings of the current study, in which both groups of responders agreed that informed consent is required before the use tele-orthodontics to inform the patients about the risks of breaching confidentiality.

A strength of the current study is that the survey tool was validated by 12 professional consultants and academic professors in orthodontics in the United States, who agreed on the content of the questions prior to survey distribution. Furthermore, this is the first study to survey orthodontists regarding their perceptions of the utilization and clinical applications of tele-orthodontics. A limitation of this study was the low response rate despite the multiple distributions. Almost all respondents who started the survey completed it within an average time of 10 min, so the questionnaire content and length were not disincentives. However, much evidence exists that clinicians generally do not respond as well to online surveys as they do to conventional paper surveys [34–37]. Additionally, the literature has shown that younger doctors are more receptive to online questionnaires [35, 37, 38], while in our sample, the mean age of responders was (54.0 ± 11.8) years, which should also be considered regarding the generalizability of the findings. A positive aspect is that online questionnaires are often answered completely and accurately [34]. Furthermore, self-selection bias may limit the applicability of our findings to a broader population, particularly those who hold negative views or have not yet engaged with tele-orthodontic options.

## Conclusions

The utilization of tele-orthodontics prevalent among AAO members, with video calls being the most commonly used tele-orthodontic communication mode.

The users of the system were more in agreement with statements regarding the efficacy of the system for orthodontic patients and in clinical practice.

Respondents indicated that both adults and children can greatly benefit from tele-orthodontics, with the greatest percentage indicating the usefulness of the system in monitoring clear aligner therapy.

Potential concerns were primarily related to breach of patients’ confidentiality and the necessity to obtain an informed consent before the use of tele-orthodontics.

## Data Availability

The data underlying this article are available in the article and in its online material.

## Appendix: Survey questions to assess orthodontists’ utilization and perceptions of tele-orthodontics

**General questions**

- Age:
- Gender:
- Years of clinical experience:
- Society of practice:
- Type of practice: Academic, Corporate, Private Practice (two or more orthodontists), Private practice (one orthodontist)
- I started using tele-orthodontics in my practice:
  1. Pre-pandemic
  2. During the pandemic (first 3–6 months until currently)
  3. During the pandemic (6 months until currently)
  4. Have not used it at all
- My preferred tele-orthodontic protocol (tick all that apply):
  1. Video calls (Zoom Video Communications, Inc; WebEx Communications, Inc.)
  2. Smile Consult by Align Technology Inc.
  3. Sharing of intraoral and extraoral photos and instant messaging
  4. Other applications

**Orthodontists’ perception of usefulness and efficacy of tele-orthodontics for patients**

- Tele-orthodontics is well-received, convenient and easy for patients

Strongly agree, agree, neutral, disagree, strongly disagree

- Tele-orthodontics can enhance guidance, advice and instructions given to the patients and ease communication with the provider

Strongly agree, agree, neutral, disagree, strongly disagree

- Tele-orthodontics reduces unnecessary visits to the clinic

Strongly agree, agree, neutral, disagree, strongly disagree

- Tele-orthodontics can assist in patient orthodontic education

Strongly agree, agree, neutral, disagree, strongly disagree

- Tele-orthodontics may act as a reminder to maintain proper oral hygiene and follow appliance instructions

Strongly agree, agree, neutral, disagree, strongly disagree

**Orthodontists’ perception of usefulness and efficacy of tele-orthodontics in clinical practice**

- Tele-orthodontics in clinical practice can be regarded as:

Extremely useful, useful, neutral, non-useful

- Tele-orthodontics was an effective mean during the pandemic in monitoring patients and facilitating communication in clinical practice

Strongly agree, agree, neutral, disagree, strongly disagree

- Tele-orthodontics helps reduce inappropriate referral rate (mild malocclusion, referral too early, and poor oral hygiene) from general practitioners and other specialists

Strongly agree, agree, neutral, disagree, strongly disagree

- Tele-orthodontics would be too expensive to set up and implement in my practice

Strongly agree, agree, neutral, disagree, strongly disagree

- There should be a charge to the patient or their insurance for an orthodontic visit using tele-orthodontics

Strongly agree, agree, neutral, disagree, strongly disagree

**Clinical applications of tele-orthodontics**

- Patients that would benefit the most from tele-orthodontics:
  1. Adults
  2. Children
  3. Both
- Tele-orthodontics is mostly used in (tick all that apply):
  1. Clear aligner therapy
  2. Emergencies
  3. Retainers
  4. Fixed orthodontic appliances
  5. Other
- Tele-orthodontics provides adequate diagnostic information most of the times and is an adequate screening tool for prospective patients

Strongly agree, agree, neutral, disagree, strongly disagree

- Tele-orthodontics helps monitor rate of tooth movement and treatment progress

Strongly agree, agree, neutral, disagree, strongly disagree

- Tele-orthodontics can be an effective tool for interceptive orthodontic treatment (using headgear, facemask, functional appliances, expanders, habit breaking appliances, bite planes, holding arches, etc.)

Strongly agree, agree, neutral, disagree, strongly disagree

- Rank the efficiency of telematic appointments from 1 (most effective) to 5 (least effective):
  1. Screening, planning, consultation
  2. Delivery and monitoring of aligners
  3. Delivery of elastics and their use
  4. Follow up appointments for visualization of tooth movement (Dental Monitoring)
  5. Handling emergencies
- There will be a significant increase in tele-orthodontics in the future

Strongly agree, agree, neutral, disagree, strongly disagree

**Limitations and concerns related to the use of tele-orthodontics**

- The irregular in-person visits may influence patients’ compliance in terms of oral hygiene maintenance and adherence to the planned treatment protocols

Strongly agree, agree, neutral, disagree, strongly disagree

- Patients with systematic conditions (obesity, hyperglycemia, diabetes, etc.), severe caries or periodontal disease and those on medications (bisphosphonates, corticosteroids, NSAID) that would jeopardize orthodontic treatment should never be candidates for tele-orthodontics

Strongly agree, agree, neutral, disagree, strongly disagree

- The biomechanical concerns of how treatment progresses would make me cautious while using remote monitoring

Strongly agree, agree, neutral, disagree, strongly disagree

**Concerns about security, confidentiality, and consent**

- Is tele-dentistry legally acceptable in your state?

Yes, no, I don’t know

- Tele-orthodontics may violate patients’ privacy and confidentiality when data is sent online

Strongly agree, agree, neutral, disagree, strongly disagree

- Informed consent is a must before the use of tele-orthodontics, as there is a risk of breaching confidentiality when patients’ photographs and other clinical information are shared

Strongly agree, agree, neutral, disagree, strongly disagree

## Abbreviations

AAO: American Association of Orthodontists
ADA: American Dental Association
CVR: Content validity ratio
DM: Dental monitoring
HIPAA: Health Insurance Portability and Accountability Act
mHealth: Mobile Health
RPM: Remote Patient Monitoring
